# Expanding the therapeutic armamentarium for advanced hepatocellular carcinoma: successful transarterial chemoembolization of peritoneal extrahepatic metastasis

**DOI:** 10.1007/s12328-024-01990-3

**Published:** 2024-05-28

**Authors:** Nicolò Brandi, Emanuela Giampalma, Matteo Renzulli

**Affiliations:** 1 Department of Radiology, Alma Mater Studiorum University of Bologna, 40138 Bologna, Italy; 2 Department of Radiology, AUSL Romagna, 48018 Faenza, Italy; 3 Radiology Unit, Morgagni-Pierantoni Hospital, AUSL Romagna, Forlì, Italy; 4 Department of Radiology, IRCCS Azienda Ospedaliero-Universitaria di Bologna, Via Albertoni 15, Bologna, Italia

**Keywords:** Hepatocellular carcinoma, Interventional radiology, Transarterial chemoembolization, Metastasis, Peritoneal

## Abstract

Hepatocellular carcinoma (HCC) is predominantly known for its intrahepatic manifestations, yet extrahepatic dissemination, particularly intraperitoneal, remains rare. Herein, we present the very first case of successful transarterial chemoembolization (TACE) for an extrahepatic peritoneal HCC nodule. This intervention underscores the potential efficacy of TACE as a viable alternative to surgery in cases where arterial vessels supplying the lesion do not nourish vital parenchymal organs.

## Introduction

Hepatocellular carcinoma (HCC) ranks sixth globally among cancers and is the third leading cause of cancer-related death [1]. While HCC typically spreads within the liver, extrahepatic dissemination is uncommon and especially occurs in patients with advanced disease [2]. The most common site of extrahepatic metastasis is the lung, followed by intra-abdominal lymph nodes, bones, and adrenal glands, whereas peritoneum is rarely involved. In particular, peritoneal dissemination can occur as a result of a ruptured HCC that spills tumor cells into the peritoneal cavity or as a complication of diagnostic and therapeutic percutaneous procedures and laparoscopic hepatectomy [3].

Treatment options for extrahepatic implants are limited, and systemic therapy is recommended by most international guidelines. Despite some studies suggesting that surgical removal of peritoneal HCC implants might improve the long-term prognosis of selected patients [4–6], only a minority of them are eligible for surgery, and even fewer for a re-excision; moreover, surgery is still an aggressive and invasive procedure, associated with nonnegligible risk and possible complications [7]. Since intraperitoneal metastases are radiologically hypervascular like primary hepatic masses, intra-arterial local therapy such as transarterial chemoembolization (TACE) can represent an effective alternative to surgery.

## Case report

A 78 year-old male with Child-Pugh B cirrhosis and chronic hepatitis C treated with sofosbuvir and velpatasvir underwent his annual follow-up at our Center. Two years prior, the patient successfully underwent radiofrequency ablation (RFA) for an HCC nodule located in segment 5. Contrast-enhanced CT demonstrated no signs of disease recurrence at the site of the previous treatment and the absence of significant ascites. However, it revealed two nodular lesions of 13 mm and 10 mm within segment 6. These lesions exhibited arterial hypervascularization and portal-venous washout consistent with HCC. In addition, a 5 mm nodular lesion was detected in the peritoneum along the right lateroconal fascia, raising suspicion of extrahepatic HCC metastasis, possibly secondary to tumor spillage following the prior percutaneous therapeutic procedure. Laboratory results indicated elevated levels of both total bilirubin (2.6 mg/dL) and albumin (3.0 g/dL), with an international normalized ratio (INR) of 1.5 and alpha-fetoprotein (AFP) levels of 16.5 ng/mL.

Both intrahepatic nodules were not suitable for ablation, and surgical resection was deemed high risk. Therefore, TACE was proposed as an alternative treatment after multidisciplinary deliberation. The treatment of the suspected extrahepatic metastasis was postponed, pending the outcome of the intra-arterial procedure.

**Fig. 1:**
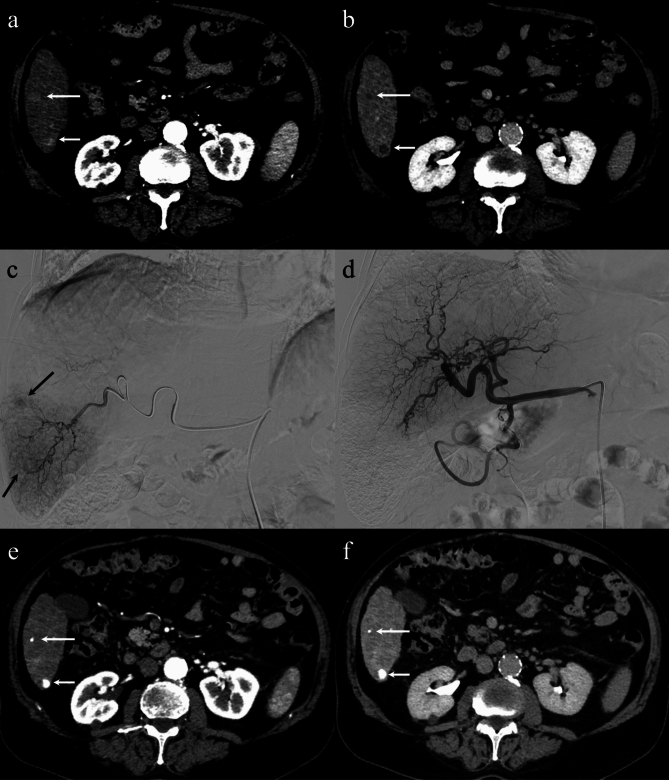
Contrast-enhanced computed tomography (CT) imaging shows the two nodular lesions of 13 mm and 10 mm within segment 6 of the liver, exhibiting arterial hypervascularization (arrows in **a**) and portal-venous washout (arrows in **b**), consistent with hepatocellular carcinoma (HCC). The angiographic study, in the parenchymal phase, confirms the two hypervascular nodules (arrows in **c**). After transarterial chemoembolization (TACE), the occlusion of the tumor-feeding vessels is demonstrated together with the patency of the remaining hepatic arteries (**d**). Contrast-enhanced CT performed 3 months post-TACE demonstrates the homogeneous accumulation of the Lipiodol in both lesions, with no signs of relapse in the arterial and portal-venous phases (arrows in **e** and **f**)

**Fig. 2:**
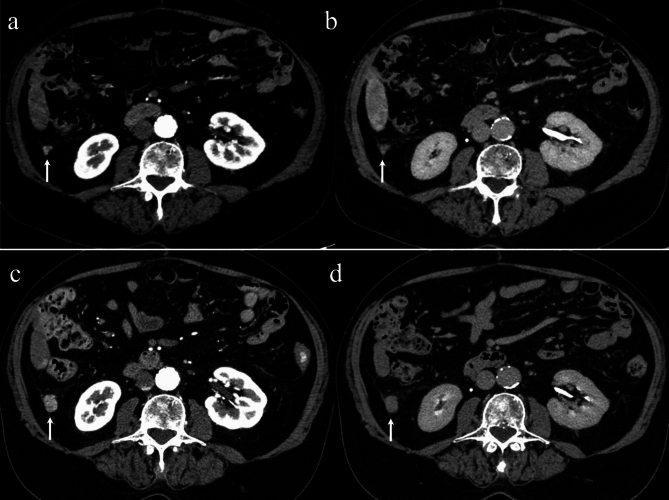
Contrast-enhanced computed tomography (CT) imaging shows a 5 mm peritoneal nodular lesion along the right lateroconal fascia in both the arterial (arrow in **a**) and portal-venous (arrow in **b**) phases. The nodule may represent an extrahepatic HCC metastasis possibly secondary to tumor seeding following a prior radiofrequency ablation (RFA). Contrast-enhanced CT performed 3 months after the transarterial chemoembolization of the two intrahepatic nodules demonstrates a notable growth of the extrahepatic deposition (10 mm vs. 5 mm) (arrows in **c** and **d**)

Under fluoroscopic guidance, TACE was performed by selectively catheterizing the hepatic arteries feeding the lesions using a highly flexible coaxial microcatheter (2.7–2.8 Fr ProgreatTM, Terumo®), passed through a 4 Fr catheter previously placed in the hepatic artery. Subsequently, a mixture of Epirubicin (Farmorubicin; Pfizer®, Latina, Italy) and iodized oil (Lipiodol; Guerbet^®^, Milan, Italy) was injected, followed by embolizing Spongel particles (Gelitaspongel^®^). Follow-up CT at 3 months post-TACE demonstrated a complete radiological response of both nodules, with homogeneous Lipiodol uptake and the disappearance of intra-tumoral enhancement (Fig. 1). However, the extrahepatic nodule exhibited notable growth (10 mm vs. 5 mm) (Fig. 2).

Considering the excellent response achieved with TACE for the two intrahepatic HCC nodules, alongside the persistent contraindications to surgical intervention, multidisciplinary consensus favored attempting TACE treatment for the extrahepatic nodule. Preliminary evaluation of maximum intensity projection (MIP) of CT images allowed accurate delineation of the tumor-feeding vessel, which originated from a thin branch of the right renal artery. Under fluoroscopic guidance, superselective catheterization of the feeding artery was performed and confirmed by cone-bean CT. In addition, the artery’s limited supply to the abdominal parenchyma and intestine was demonstrated. Finally, a mixture of epirubicin (Farmorubicin; Pfizer^®^, Latina, Italy; 50 mg) and iodized oil (Lipiodol; Guerbet®, Milan, Italy; 10 ml) was injected, followed by embolization using Glubran II particles (GEM Italy^®^) until complete blockage of the vessel was demonstrated. The patient tolerated the procedure well without any immediate complications.

**Fig. 3:**
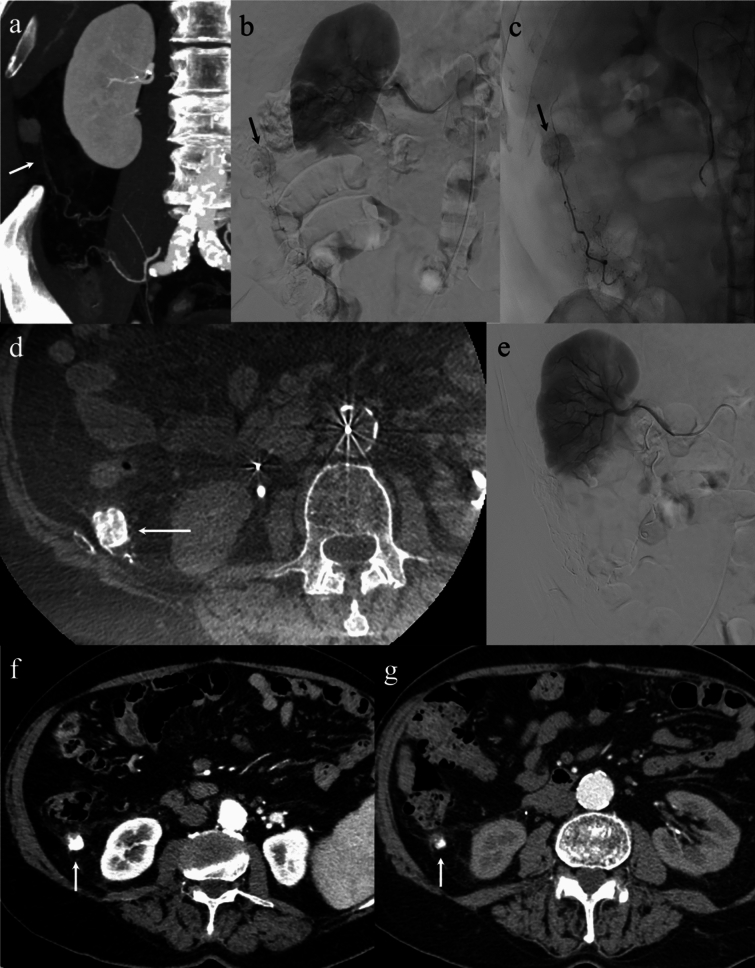
Preliminary evaluation of maximum intensity projection (MIP) of computed tomography (CT) images allowed accurate delineation of the tumor-feeding vessel of the peritoneal metastasis from hepatocellular carcinoma (HCC), which originated from a thin branch of the right renal artery (arrow in **a**). The angiographic study confirmed the blood supply of the extrahepatic metastasis and its rich blood supply similar to its counterparts in the liver (arrow in **b** and **c**). The cone-beam computed tomography (CT) also demonstrated the superselective catheterization of this thin tumor-feeding artery and its limited supply to the abdominal parenchyma and intestine (arrow in **d**). After transarterial chemoembolization (TACE), the occlusion of the tumor-feeding vessel is demonstrated together with the patency of the remaining hepatic arteries (**e**). Contrast-enhanced CT performed 1-month post-TACE revealed successful therapeutic agent delivery and the disappearance of arterial enhancement (arrow in **f**). The Lipiodol deposition was reduced at the 12-month follow-up, and the lesion demonstrated a significant dimensional regression (arrow in **g**)

Follow-up imaging at 1 month post-TACE revealed successful therapeutic agent delivery and the disappearance of arterial enhancement. The Lipiodol deposition was reduced at the 12 month follow-up, and the lesion demonstrated a significant dimensional regression (Fig. 3).

## Discussion

Although rare, extrahepatic HCC spread poses significant challenges as patients are considered at an advanced stage, limiting treatment to systemic options [8]. Several studies have demonstrated that resection of peritoneal metastases may benefit select patients, particularly those with absent or well-controlled intrahepatic disease, improving their long-term survival [4–6]. Nonetheless, eligibility is low (36%), and even lower for a re-excision, and the early recurrence rate is still high (66.6%) [9]; additionally, the procedure carries nonnegligible risks and possible complications. Therefore, there is a need for alternative non-surgical treatment modality that minimizes side effects while still improving both quality of life and survival rates for patients with advanced HCC and peritoneal metastases.

In the present case, TACE emerged as a novel and effective intervention for an extrahepatic peritoneal HCC nodule. In particular, since metastatic tumors are rich in blood supply similar to their counterparts in the liver, TACE can represent a valuable therapeutic tool, offering advantages over surgery by avoiding associated morbidity. In addition, the procedure can be repeated as needed to achieve a complete response and/or treat other metastases. TACE is particularly compelling in the setting of singular extrahepatic metastases or a limited number of implants, as can occur in tumor spillage following invasive diagnostic and/or therapeutic procedures, similar to what probably happened in the present case and is reported to happen in 3.17% and 1.73% of patients following biopsy and RFA, respectively [10]. Indeed, although these patients are at an advanced stage by definition, their prognosis is not comparable to that of HCC patients experiencing extrahepatic spread as a natural progression of an uncontrolled disease.

Recent advancements in imaging techniques and microsphere technology have greatly improved the safety and efficacy of locoregional intra-arterial therapies, allowing TACE to be extended beyond its conventional indications. In this particular case, the success of TACE treatment for extrahepatic metastasis was attributed to several factors. The nodule was solitary, and its feeding artery was clearly identified during the preliminary evaluation of MIP images, with minimal risk of inducing bowel ischemia. In addition, both intrahepatic nodules were successfully treated with TACE, achieving a complete radiological response and resulting in no signs of active disease in the liver. Finally, the extrahepatic implant was likely a complication from a previous percutaneous procedure rather than a result of the extrahepatic spread of the primary disease; this distinction led to a more favorable prognosis compared to patients with actual advanced disease. Nonetheless, this procedure may not be a viable option in all clinical scenarios and still requires careful evaluation before proceeding, ensuring an adequate artery size for catheterization and the absence of blood supply to vital organs. In some cases, in fact, systemic therapy may be the only feasible treatment choice. Therefore, interdisciplinary collaboration is pivotal in navigating complex clinical scenarios to achieve the best personalized approach for patients with advanced HCC.
